# Nasopharyngeal microbiota profiling of pregnant women with SARS-CoV-2 infection

**DOI:** 10.1038/s41598-022-17542-z

**Published:** 2022-08-04

**Authors:** Francesca Crovetto, Marta Selma-Royo, Fàtima Crispi, Belén Carbonetto, Rosalia Pascal, Marta Larroya, Irene Casas, Marta Tortajada, Nuria Escudero, Carmen Muñoz-Almagro, Maria Dolores Gomez-Roig, Pedro González-Torres, Maria Carmen Collado, Eduard Gratacos

**Affiliations:** 1 Department of Maternal-Fetal Medicine, BCNatal, Barcelona Center for Maternal-Fetal and Neonatal Medicine, Hospital Sant Joan de Déu and Hospital Clínic, Universitat de Barcelona, Passeig de Sant Joan de Déu 2, 08950 Esplugues de Llobregat, Barcelona, Spain; 2 Institut de Recerca Sant Joan de Deu, Barcelona, Spain; 3 Primary Care Interventions to Prevent Maternal and Child Chronic Diseases of Perinatal and Developmental Origin (RICORS), Instituto de Salud Carlos III, Madrid, Spain; 4 Institute of Agrochemistry and Food Technology (IATA-CSIC), National Research Council, Agustin Escardino 7, 46980 Paterna, Valencia Spain; 5 Institut de Recerca August Pi Sunyer, Barcelona, Spain; 6 Center for Biomedical Network Research on Rare Diseases, Barcelona, Spain; 7 Microomics Systems S. L, Barcelona, Spain; 8 Ciber of Epidemiology and Public Health (CIBERESP), Madrid, Spain; 9 Department of Medicine, Universitat Internacional de Catalunya, Barcelona, Spain

**Keywords:** Clinical microbiology, SARS-CoV-2

## Abstract

We aimed to analyze the nasopharyngeal microbiota profiles in pregnant women with and without SARS-CoV-2 infection, considered a vulnerable population during COVID-19 pandemic. Pregnant women were enrolled from a multicenter prospective population-based cohort during the first SARS-CoV-2 wave in Spain (March-June 2020 in Barcelona, Spain) in which the status of SARS-CoV-2 infection was determined by nasopharyngeal RT–PCR and antibodies in peripheral blood. Women were randomly selected for this cross-sectional study on microbiota. DNA was extracted from nasopharyngeal swab samples, and the V3-V4 region of the 16S rRNA of bacteria was amplified using region-specific primers. The differential abundance of taxa was tested, and alpha/beta diversity was evaluated. Among 76 women, 38 were classified as positive and 38 as negative for SARS-CoV-2 infection. All positive women were diagnosed by SARS-CoV-2 IgG and IgM/IgA antibodies, and 14 (37%) also had a positive RT–PCR. The overall composition of the nasopharyngeal microbiota differ in pregnant women with SARS-CoV-2 infection (positive SARS-CoV-2 antibodies), compared to those without the infection (negative SARS-CoV-2 antibodies) (*p* = 0.001), with a higher relative abundance of the Tenericutes and Bacteroidetes phyla and a higher abundance of the *Prevotellaceae* family. Infected women presented a different pattern of microbiota profiling due to beta diversity and higher richness (observed ASV < 0.001) and evenness (Shannon index < 0.001) at alpha diversity. These changes were also present in women after acute infection, as revealed by negative RT–PCR but positive SARS-CoV-2 antibodies, suggesting a potential association between SARS-CoV-2 infection and long-lasting shift in the nasopharyngeal microbiota. No significant differences were reported in mild vs. severe cases. This is the first study on nasopharyngeal microbiota during pregnancy. Pregnant women with SARS-CoV-2 infection had a different nasopharyngeal microbiota profile compared to negative cases.

## Introduction

The upper respiratory tract is the major portal of entry for infectious droplets or aerosol-transmitted microorganisms. The barrier function of its mucosa and the regulation of the immune response are modulated by the microbiota, the communities of microorganisms that colonize all of the surfaces of the human body, participating in host physiological and pathological processes^1^. Evidence suggests that dysbiosis of the upper respiratory tract (nose and nasopharynx) microbiota modulates the host´s susceptibility to pathological conditions, such as acute respiratory tract infections^2,3^.

Severe acute respiratory syndrome coronavirus 2 (SARS-CoV-2) is transmitted through microdroplets and aerosols produced by sneezing, coughing, or speaking^4^. The virus penetrates the host through the upper airways, which represent the first defense to avoid infection. The microbiota of the respiratory system may play a role from initiation to progression of coronavirus disease (COVID-19)^5^. However, evidence on the relationship between the upper respiratory tract microbiota and SARS-CoV-2 infection is still scarce and discordant. A study on 56 COVID-19 patients reported differences in the composition of specific operational taxonomy units (OTUs), mostly belonging to Bacteroidetes and Firmicutes phyla, and a loss of complexity abundance networks in the most severe cases^6^. Similarly, other studies demonstrated differences in the Chao1 and Shannon indexes^7^, with an age dependency of the pharyngeal profile^8^. In contrast, other authors could not find any differences in either bacterial richness/diversity or composition^9–11^, even if patients with overt COVID-19 had a lower abundance of Proteobacteria and Fusobacteria phyla^10^ and lower taxonomic richness^12^.

Pregnancy is a unique physiological state in which all body systems participate, including hormonal, immune and metabolic pathways^13^. Recent evidence illustrated gut microbiota changes over the course of a healthy pregnancy^14^; nevertheless, other human niches with potential physiological effects have been poorly studied. SARS-CoV-2 infection during pregnancy is mostly asymptomatic or mild^15–17^, but similar to other respiratory viruses, there is a greater risk of severe respiratory complications compared with nonpregnant women, particularly in late gestation^18^. The characteristics of nasopharyngeal microbiota in women with SARS-CoV-2 infection during pregnancy have not been investigated.

In this study, we aimed to study the impact of SARS-CoV-2 infection on the nasopharyngeal microbiota of pregnant women at the third trimester of pregnancy. We further investigated the potential differences in nasopharyngeal microbiota in women with active *versus* past infection determined by SARS-CoV-2 PCR or the presence of specific viral antibodies; as well as the asymptomatic *versus* symptomatic SARS-CoV-2 infection symptoms and also, in relation to antibody titers.

## Methods

### Study design

Pregnant women were selected from a large multicenter prospective population-based cohort study conducted from March 15 to May 31, 2020, in Barcelona, Spain^17^, during the first SARS-CoV-2 wave in Spain: all women consecutively admitted in three hospitals for delivery were recruited. Nasopharyngeal swab detection of SARS-CoV-2 RNA by real-time polymerase chain reaction (RT–PCR) and microbiota study and peripheral blood for antibody detection were obtained in all participants at recruitment. All the women consecutively admitted in the hospitals were tested for SARS-CoV-2 infection and those that accepted to participate in the study were assigned to the positive or negative group according to the result of both RT-PCR and serological test (see laboratory diagnostic procedures for SARS-CoV-2 infection section). For this specific study focused on nasopharyngeal microbiota, 76 women were randomly selected from the prospective cohort^17^ to study the nasopharyngeal microbiota (half of them positive, half negative).

The study was approved by the ethics committee at each of the three involved institution (Ethical Committee of Hospital Clínic, study number HCB/2020/0434, Ethical Committee of Hospital Sant Joan de Déu study number PIC-56-20), and informed written consent was obtained from all women.

All methods were carried out in accordance with relevant guidelines and regulations involving human participants and human samples were conducted in accordance with the 1964 Helsinki Declaration and its later amendments.

### Data collection

Pregnancy and perinatal data were obtained from electronic medical records. COVID-19 symptoms were recorded using a structured questionnaire for all pregnant women, which included questions about risk factors and about any symptom suggestive of COVID-19 noticed between mid-February 2020 and the time of testing for SARS-CoV-2.

### Sample collection and laboratory diagnostic procedures for SARS-CoV-2 infection

For each participant, nasopharyngeal swab samples for SARS-CoV-2 RNA RT–PCR were collected by the hospital’s trained staff. Samples were collected in storage tubes (Micronics) with Zymo DNA/RNA Shield Lysis Buffer. RNA was extracted using the Quick-DNA/RNA Viral MagBead kit (Zymo) and the TECAN Dreamprep robot. Five microliters of RNA solution were added to 15 μl of rRT-PCR master mix (Luna Universal Probe One-Step RT–qPCR Kit; New England Biolabs, USA) and used for amplification of SARS-CoV-2 N1 and N2 regions, as well as the human RNase P gene as a control, as described in the CDC-006-00019 CDC/DDID/NCIRD/Division of Viral Diseases protocol released 3/30/2020. A SARS-CoV-2-positive result was considered if the Ct values for N1, N2 and RNase P were below 38. Samples discordant for N1 and N2 were repeated, and samples with a Ct ≥ 40 for RNase P were considered invalid.

Blood samples were drawn from peripheral veins for each participant. Serum was separated by centrifugation at 1500*g* for 10 min at 4 °C, and samples were immediately stored at − 80 °C until processing. SARS-CoV-2 IgG and IgM/IgA antibodies were tested in all maternal samples using COVID-19 VIRCLIA® Monotest (Vircell Microbiologist, Granada, Spain). Indeterminate results were retested (VITROS® Immunodiagnostic Products Anti-SARS-CoV2 Total Tests, Ortho Clinical Diagnostics, Rochester, NY, USA) and classified as positive or negative. Likewise, all samples that were positive for IgM + IgA but negative for IgG in women reporting no symptoms suggestive of COVID-19 during the 10 weeks prior to testing were retested by a quantitative suspension array assay based on xMAP Luminex technology (Luminex Corporation, Austin, TX, USA)^19^ and classified as positive or negative. A positive serological result was considered in the presence of any of the following: (1) seropositivity for IgG, (2) seropositivity for IgM + IgA in women with symptomatic COVID-19, or (3) seropositivity for IgM and/or IgA confirmed by two tests (Vircell and Luminex).

SARS-CoV-2 infection was defined either by positive RT–PCR in nasopharyngeal swabs or a positive serological result. Active infection was defined by a positive RT–PCR, while women with a negative RT–PCR but positive serological testing were defined as past infection. Among SARS-CoV-2-infected women, we defined as symptomatic those with at least one of the following symptoms: fever, dry cough, loss of taste or smell, dyspnea, headache, myalgias, diarrhea, sore throat and rash on skin or discoloration of fingers/toes. Thus, positive cases were subclassified as asymptomatic if no symptoms were reported, mild if there was at least one symptom compatible with the infection, or severe if symptoms suggestive of pneumonia (persistent fever and cough) or dyspnea were reported, which required hospital admission for surveillance^20^.

### DNA isolation and sequencing

DNA was extracted from nasopharyngeal swab samples using the ZymoBIOMICSTM 96 MagBead DNA Kit (Zymo Research) following manufacturer’s instructions. The extraction tubes were agitated using Tissue lyser II (Qiagen, Hilden, Germany) at 30 Hz/s for 10 min.

After DNA extraction, the V3-V4 region of the 16S rRNA of bacteria was amplified using region-specific primers (For: 5’TCGTCGGCAGCGTCAGATGTGTATAAGAGACAGCCTACGGGNGGCWGCAG-3′, Reverse 5′GTCTCGTGGGCTCGGAGATGTGTATAAGAGACAGGACTACHVGGGTATCTAATCC-3′) (ref).

Specific amplicons were obtained following the PCR protocol: 3 min at 95 °C (initial denaturation) followed by 35 cycles: 30 s at 95 °C 30 s at 55 °C, and 30 s at 72 °C, and a final elongation step of 5 min at 72 °C. PCR products were purified using AMPure XP beads (Beckman Coulter, Nyon, Switzerland) with a 0.9 × ratio according to manufacturer’s instructions. The above described primers contain overhangs allowing the addition of full-length Nextera barcoded adapters for multiplex sequencing in a second PCR step, resulting in sequencing ready libraries with approximately 450 bp insert sizes. In brief, 5 μl of the first PCR purified product were used as template for a second PCR with Nextera XT v2 adaptor primers in a final volume of 30 μl using the same PCR mix and thermal profile as for the first PCR but with only 8 cycles. 25 μl of the second PCR product were purified with SequalPrep normalization kit (Invitrogen, ThermoFisher Scientific, Waltham, MA, USA), according to the manufacturer's protocol. Mock community DNA was included as positive control for library preparation (Zymobiomics Microbial Community DNA, ZymoResearch, Irvine, CA, USA) as well as negative control to control the amplification and sequencing environmental and cross-contaminations.

Libraries were eluted in 20 μl final volume and pooled for sequencing. The final pool was quantified by qPCR using Kapa library quantification kit for Illumina Platforms (Kapa Biosystems, SigmaAldrich, Saint Louis, MO, USA) on an ABI 7900HT real-time cycler (Applied Biosystems, ThermoFisher Scientific, Waltham, MA, USA). Sequencing was performed using Illumina MiSeq (2 × 300 bp) and v3 chemistry with a loading concentration of 10 pM. In all cases, 15% of PhIX control libraries were used to increase the diversity of the sequenced sample. Negative controls included sample collection buffer, DNA extraction, and PCR amplification steps, PCR products after both PCR steps were visualized by electrophoresis gel (1.5% agarose) with SYBR Safe (ThermoFisher Scientific, Waltham, MA, USA). No visible bands were observed.

### Computational and statistical analysis

Data are presented as the mean (standard deviation, SD), median (interquartile range, IQR) or number (percentage), as appropriate. Statistical analysis for comparison of clinical and perinatal characteristics included the use of *Student’s t test* or Mann–Whitney U tests and Pearson χ^2^ test for continuous and categorical variables, respectively, to compare SARS-CoV-2-positive *vs.* SARS-CoV-2-negative women. Differences were considered significant when *p* < 0.05.

Raw demultiplexed forward and reverse reads were processed using the following methods and pipelines as implemented in QIIME2 version 2020.2 with default parameters unless stated^21^. DADA2 was used for quality filtering, denoising, paired-end merging and amplicon sequence variant calling (amplicon sequence variant, ASV, i.e., phylotypes) using the qiime *dada2 denoise-paired* method^22^. Q20 was used as a quality threshold to define read sizes for trimming before merging. Reads were truncated at the position when the 75th percentile Phred score felt below Q20: 284 bp for forward reads and 224 bp for reverse reads. The average of the amplicon size obtained was 404.09 bp (min = 261 and max = 432 m STD = 14.8) after merging of the paired end reads. Phylotypes were filtered to discard contaminant eukaryote DNA-derived amplicons using BLAST against the eukaryote database with a 90% identity cutoff. After quality filtering steps, the average sample size was 28,303 reads (min: 8,965 reads, max: 64,404X reads), and 4823 phylotypes were detected. Negative controls were used to detect environmentally derived contaminants. Taxonomic affiliation results revealed that most contaminant amplicons were either absent in most samples or were at least two orders of magnitude less abundant than in the negative control. From those detected phylotypes, 43 phylotypes assigned to 36 taxa at Level 7 (ASV) were detected in the samples. Taxa (n = 22) that presented differences between experimental groups were removed from the analysis to avoid contaminant noise from diversity and composition analyses. A complete list of these phylotypes is reported in Table S1.

ASVs were aligned using the *qiime alignment mafft* method^23^. The alignment was used to create a tree and to calculate phylogenetic relations between ASVs using the *qiime phylogeny fasttree* method^24^. ASV tables were subsampled without replacement to even sample sizes for diversity analysis using *the QIIME diversity core-metrics-phylogenetic* pipeline. The smallest sample size was chosen for subsampling⁠. Jaccard, Bray Curtis and unweighted and weighted UniFrac distances^25^ were calculated to compare community structures. Alpha diversity metrics calculated were observed OTU number (i.e., richness), Pielou's evenness index and Shannon index. Taxonomic assignment of ASVs was performed using a Bayesian classifier trained with the Silva database v.132 (i.e., 99% OTU database) using the *QIIME feature-classifier classify-sklearn* method^26^.

The differential abundance of taxa was tested using two methods, ANCOM^27^ and the Mann–Whitney nonparametric test on the relative abundance of taxa (total sum scale-TSS). Alpha diversity comparisons were performed using the Kruskal–Wallis nonparametric test. After Kruskal–Wallis, Conover’s test with FDR Benjamini–Hochberg correction was added for pairwise comparison. Beta diversity distance matrices and ASV tables were used to calculate principal coordinates (PCoA) and construct ordination plots. The significance of groups in community structure was tested using PERMANOVA^28^, and the Permdisp test was used to identify location *vs.* dispersion effects^28^. BiodiversityR version 2.11-1^29^, PMCMR version 4.3^30^, RVAideMemoire version 0.9-7^31^, vegan version 2.5-5 packages^32^, R software package version 3.6.0 and IBM SPSS 26.0 (IBM Corp. Released 2019. IBM SPSS Statistics for Windows, Version 26.0. Armonk, NY: IBM Corp) were used to conduct all the statistical analyses. Spearman correlations between IgG and IgM/IgA levels and microbial taxa were performed on the Calypso online platform^33^ and then plotted using the ggplots v 3.1.1 package^34^. Linear discriminant analysis (LDA) effect sized (LEfSe) analysis was performed for biomarker discovery using a size-effect cutoff of 3.0 on the logarithmic LDA score in the Calypso online platform. All plots were performed using the mentioned packages and ggplot2, qiime2R v. 0.99, forcats v. 0.5.1, ggpubr v. 0.4.0 and RColorBrewer v. 1.1-3 packages.

## Results

### Study population and pregnancy outcomes

The baseline characteristics of the study population are shown in Table 1. The average of maternal age was 31.3 years (SD 5.9; minimum 18.7, maximum 43.5 years). No differences were reported between positive and negative SARS-CoV-2 infected women (Table S2). The clinical characteristics of the study population according to SARS-CoV-2 infection are shown in Table 2. Among women with SARS-CoV-2 infection, 20 (52.6%) reported the presence of at least one symptom, and the most common symptoms were dry cough and fever (29% and 26%, respectively). Of a total of 38 infected women due to positive antibodies, only 14 (36.8%) also had a positive RT–PCR. Although 7 women (18%) required hospital admission for severe SARS-CoV-2 infection, only one presented pneumonia. None of them required intensive care unit (ICU) admission.

**Table 1:** Baseline characteristics of the study population.

	N = 76
Age, years	31.1 (27.3–35.8)
**Race or ethnic group**	
White	48 (63.2%)
Latin-American	15 (19.7%)
Black	0 (0%)
Asian	8 (10.5%)
Maghreb	5 (6.6%)
**Socioeconomical status**^#^	25 (32.9%)
Low	7 (9.2%)
Medium	35 (46.1%)
High	34 (44.7%)
Pre-pregnancy body mass index, kg/m^2^	24.4 ± 4.7
Smoking during pregnancy	8 (10.5%)
**Relevant comorbidities**	
Chronic hypertension	2 (2.6%)
Diabetes mellitus	2 (2.6%)
Obesity^†^	10 (13.2%)
Asthma	10 (13.2%)
Hypothyroidism	10 (13.2%)
**Pregnancy history**	
Nulliparous	43 (56.6%)
Assisted reproductive technologies	6 (7.9%)
Multiple gestation	1 (1.3%)
Gestational age at recruitment, weeks	39.5 (2.1)

Data are n (%) or median (IQR) or mean ± SD.

^#^Low socioeconomic status defined as study level (low: no studies, primary; medium: secondary; high: university).

^†^Obesity defined as body mass index > 30 kg/m^2^.

**Table 2:** Clinical characteristics of the study population subdivided according to SARS-CoV-2 infection.

	SARS-CoV-2 negative (n = 38)	SARS-CoV-2 positive (n = 38)	*p*
**Symptoms compatible with SARS-CoV-2 infection within the last 10 weeks**			
None	38 (100%)	18 (47.4%)	< 0.001
Fever ≥ 37.7 °C	0 (0%)	10 (26.3%)	< 0.001
Dry cough	0 (0%)	11 (28.9%)	< 0.001
Loss of taste or smell	0 (0%)	9 (23.7%)	0.001
Difficulty breathing or shortness of breath	0 (0%)	7 (18.4%)	0.005
Myalgia	0 (0%)	4 (10.5%)	0.040
Diarrhea	0 (0%)	3 (7.9%)	0.077
Fatigue	0 (0%)	4 (10.5%)	0.040
Sore throat	0 (0%)	1 (2.6%)	0.314
At least two symptoms or anosmia	0 (0%)	14 (36.8%)	< 0.001
At least three symptoms or anosmia	0 (0%)	13 (34.2%)	< 0.001
Presence of fever, cough and dyspnea	0 (0%)	6 (15.8%)	0.011
**Diagnosis of SARS-CoV-2 infection**			
RT-PCR positive	0 (0%)	14 (36.8%)	< 0.001
IgM/A and/or IgG for SARS-CoV-2 positive	0 (0%)	38 (100%)	< 0.001
IgM/A for SARS-CoV-2 positive	0 (0%)	26 (68.4%)	< 0.001
IgG for SARS-CoV-2 positive	0 (0%)	30 (78.9%)	< 0.001
Hospital admission for COVID-19	0 (0%)	7 (18.4%)	0.005
Pneumonia	0 (0%)	1 (2.6%)	0.314

Data are n (%).

*SARS-CoV-2* severe acute respiratory syndrome coronavirus 2 (SARS-CoV-2), *RT-PCR* reverse transcriptase polymerase chain reaction.

Pregnancy and perinatal outcomes in women depending on SARS-CoV-2 status are reported in Table S3. None of the newborns were infected by SARS-CoV-2. No differences between antibiotic consumption during pregnancy or delivery were reported.

### Nasopharyngeal microbiota in the overall study population

Pregnant nasopharyngeal microbial communities were dominated by the Firmicutes phylum (34.7 ± 8.4%), followed by Proteobacteria (26.1 ± 11.7%) and Actinobacteria (20.2 ± 10.5%) (Figure S1). At the genus level, *Corynebacterium* (14 ± 11.4%) and *Staphylococcus* (9 ± 8.2%) were the most abundant genera (Figure S1).

### SARS-CoV-2-infected pregnant women showed differences in nasopharyngeal microbiota

**Figure 1:**
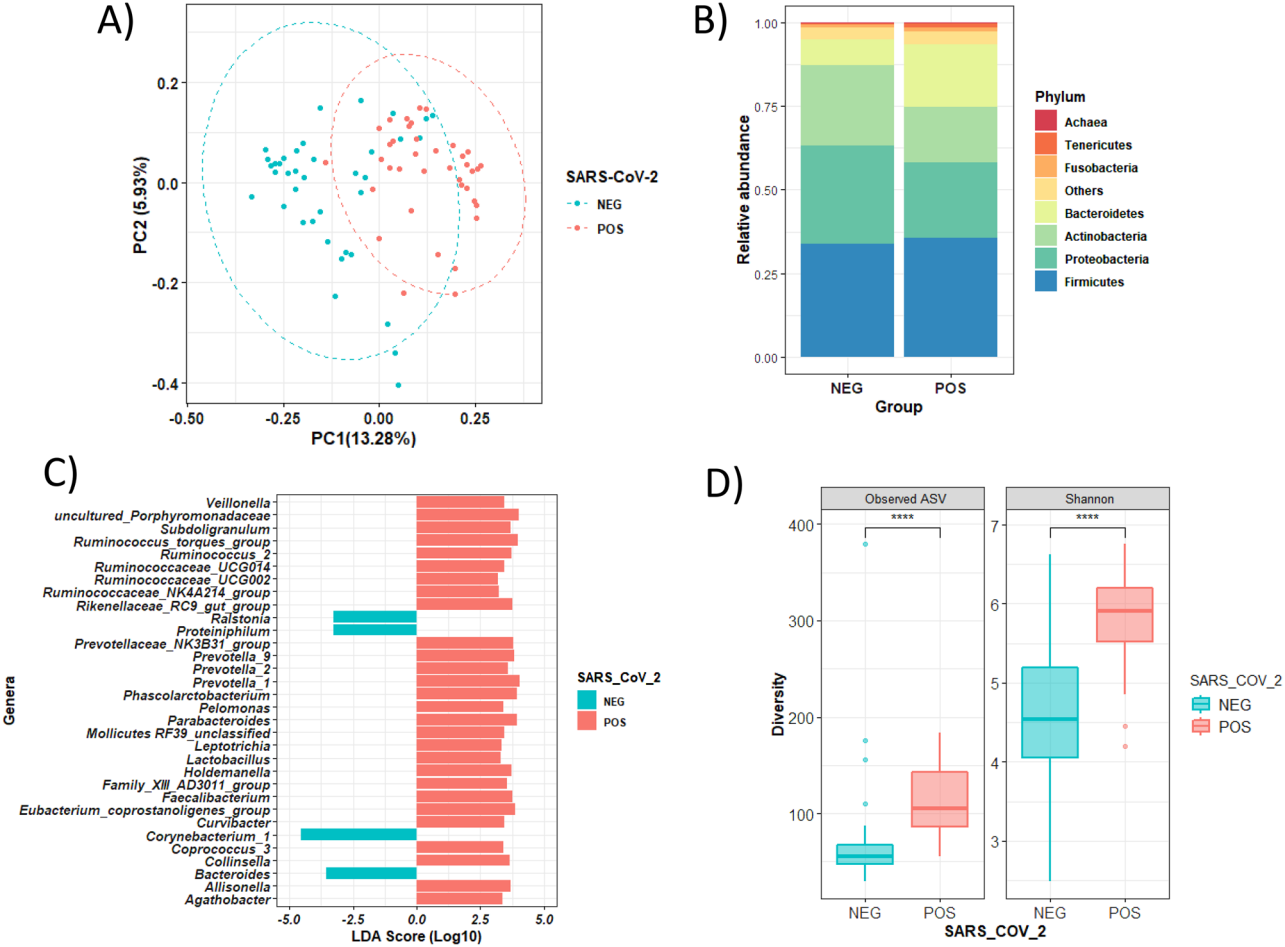
The nasopharyngeal microbiota of pregnant women is altered by SARS-CoV-2 infection. (**A**) Principal coordinates analysis (PCoA) ordination plot based on unweighted UniFrac distances according to SARS-CoV-2 infection. Each point corresponds to a sample. (**B**) Barplots showing the composition of the nasopharyngeal microbiota of the population in healthy (NEG) and SARS-CoV-2-infected (POS) pregnant women. Phyla with a relative abundance lower than 0.5% and Cyanobacteria were grouped as “Others” for plotting. (**C**) LDA effect size (LEfSe) analysis showing the genera that most discriminate both health conditions (infected *vs.* no infected). An LDA score > 3 was considered a significant threshold. (**D**) Boxplots showing the differences in the alpha diversity measured as observed ASV (amplicon sequence variant) and Shannon indexes according to SARS-CoV-2 infection. Statistical analysis of the differences between groups was calculated using the Kruskal–Wallis test with FDR correction for multiple comparisons. *POS* Positive result for SARS-CoV-2 (red), *NEG* negative result for SARS-CoV-2 (blue). *p < 0.05; **p < 0.01; ***p < 0.001.

The structure of the nasopharyngeal microbial population was different between pregnant women with and without SARS-CoV-2 infection (Adonis based on unweighted UniFrac distance, F = 1.36, *p* = 0.001) (Fig. 1A). To further explore which taxa were specifically related to the infection, the ANCOM test identified 2 phyla and 18 genera whose relative abundance differed between positive and negative cases (Fig. 1B, C). At the phylum level, the microbiota of infected women was enriched in members of the Bacteroidetes and Tenericutes phyla (Fig. 1B, Table S4). At the genus level, microbial shifts were related to an increase in the relative abundance of several groups from the *Prevotellaceae* family, including the *Prevotellaceae NK3B31 group*, *Prevotella_1*, *Prevotella_9* and unclassified ASV from this family (Table S5). In addition, other genera from the *Ruminococcaceae* and *Lachnospiraceae* families, such as *Ruminococcaceae UCG-014*, *Ruminococcus 2*, *Ruminococcus torques* group, *Suboligranumlum* and *Faecalibacterium,* were also found to be enriched in SARS-CoV-2-infected pregnant women compared to uninfected women. Regarding the phylum Bacteroidetes, uninfected women displayed a lower relative abundance of the *Porphyromonadaceae* uncultured genus, *Parabacteroides* and *Rikenellaceae* RC9 group than those who were infected. These genera were also overrepresented in infected nasopharyngeal women, as reported by LEfSe analysis (Fig. 1C).

Alpha diversity analysis showed that SARS-CoV-2-infected pregnant women harbored a higher number of observed ASVs (*p* < 0.001) and a higher Shannon diversity index (*p* < 0.001) in their nasopharyngeal microbial populations than negative women (Fig. 1D). Indeed, Bacteroidetes and Tenericutes phyla, both enriched in nasopharyngeal microbiota of infected women, were also positive associated with higher alpha diversity indexes (Spearman correlation *p* < 0.05) (Figure S2), while lower abundance of Actinobacteria phylum was related to both, higher Shannon index (rho =  − 0.58, *p* < 0.001*, q* < 0.001) and higher number of Observed ASV (rho =  − 0.34, *p* = 0.003*, q* = 0.004) indexes, mainly due to the negative relation of *Corynebacterium* genus to both diversity indexes (not shown).

### Differences in nasopharyngeal microbiota persist in women with a past SARS-CoV-2 infection

**Figure 2:**
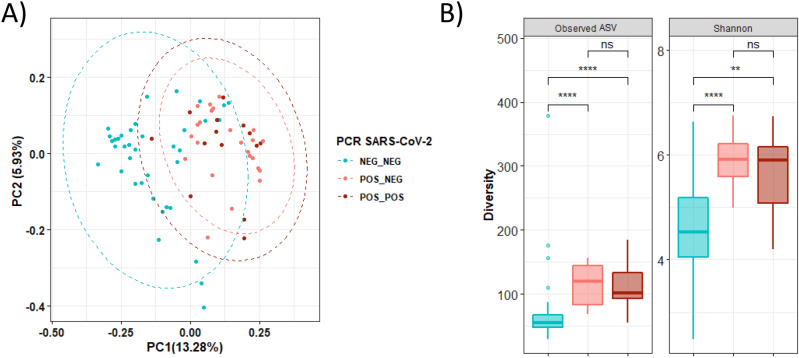
Similar nasopharyngeal microbiota in SARS-CoV-2-infected pregnant women with an active infection (positive RT–PCR and antibodies) *versus* a past infection (negative RT–PCR but positive antibodies). (**A**) Principal coordinates analysis (PCoA) ordination plot based on unweighted UniFrac distances according to the results of both serological and RT–PCR tests for SARS-CoV-2 infection (ADONIS F = 1.36*, p* = 0.001). Each point corresponds to a sample. (**B**) Boxplots showing the differences in the alpha diversity measured as observed ASV and Shannon indexes according to the results of both serological and RT–PCR tests for SARS-CoV-2 infection. POS_POS: Pregnant women with SARS-CoV-2 positive result for serological test and for nasopharyngeal RT–PCR (active infection); POS_NEG: SARS-CoV-2 positive result for serological test but negative nasopharyngeal RT–PCR (past infection); NEG-NEG: Noninfected pregnant women with SARS-CoV-2 negative serological and nasopharyngeal RT–PCR results. Statistical analysis of the differences between groups was calculated using the Kruskal–Wallis test with FDR correction for multiple comparisons. *p < 0.05; **p < 0.01; ***p < 0.001; ****p < 0.0001.

To gain more insight into the duration of the alterations in nasopharyngeal microbiota related to SARS-CoV-2 infection, we performed a comparison between SARS-CoV-2-infected mothers with an active infection (positive result in the RT–PCR at sample collection) and those with a past infection (negative result in the RT–PCR but positive antibodies) (Fig. 2). The effect of viral infection on both alpha and beta diversity was comparable in those women with a past infection, as both infected populations showed a similar nasopharyngeal microbiota profile. Beta-diversity analysis based on the unweighted UniFrac distance showed that women with a SARS-CoV-2 infection were clustered together independently of their infectious status at recruitment (Fig. 2A). Indeed, only the *Deinococcus-Thermus* (*q* = 0.021) phylum, with minor representation in nasopharyngeal microbiota, was enriched in women with a past infection. The phylum Actinobacteria was diminished in mothers with a past infection compared to control women (*q* = 0.007), suggesting the possible alteration of the nasopharyngeal microbiota after viral infection (Figure S3). No significant differences were observed at the genus level. Furthermore, while women who were negative for SARS-CoV-2 infection showed significantly lower diversity (observed ASV and Shannon index) than both women with a past infection (*q* =  < 0.001 and *q* < 0.001*,* respectively) and those with an active infection (*q* < 0.001*; q* = 0.001), no differences were found between infected groups (*q* = 0.964*; q* = 0.545) (Fig. 2B).

### SARS-CoV-2 antibody concentrations are associated with shifts in nasopharyngeal microbiota composition

**Figure 3:**
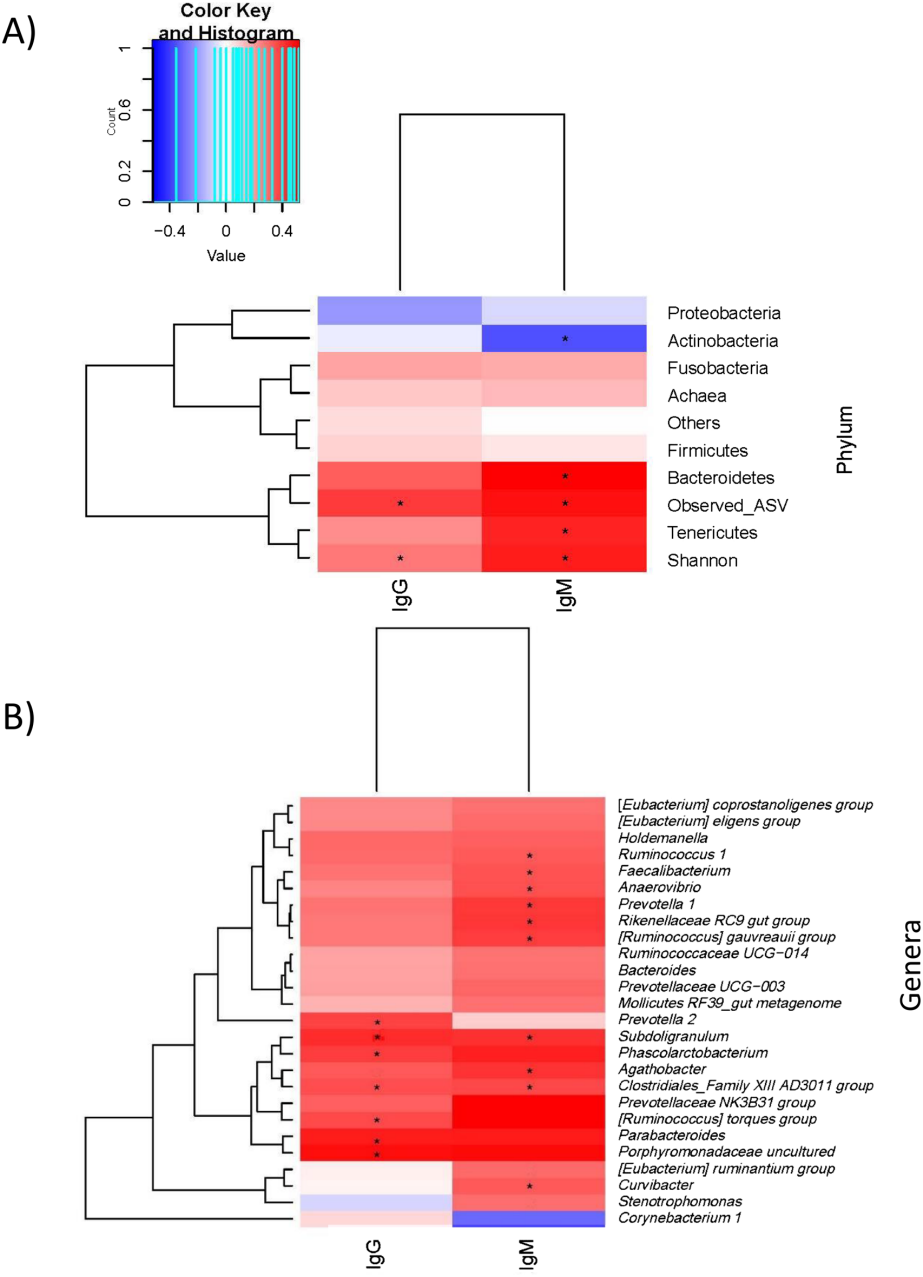
Several taxa of nasopharyngeal microbiota from pregnant women were related to the concentrations of both immunoglobulin M/A and G. Heatmap of the Spearman correlations between microbial taxa in the nasopharyngeal microbiome of pregnant women at the phylum (**A**) and genus levels (**B**) and the plasma concentration of immunoglobulin M/A (IgM/IGA) and G (IgG). At the phylum level, those phyla with a relative abundance lower than 0.5 and Cyanobacteria were grouped as “Others”. Only those genera with significant associations with at least one of the analyzed immunoglobulins are shown. The significant associations are marked with an asterisk. The color of the cell represents the positive (red) or negative (blue) association.

Both IgG and IgM/A levels were associated with the pregnant nasopharyngeal microbiota (Fig. 3). In terms of alpha diversity, both IgG and IgM/A demonstrated a positive correlation (*p* < 0.001) with diversity indexes (Fig. 3A). Furthermore, a positive relation was observed between IgM/A and the proportion of Bacteroidetes (rho = 0.52, *p* < 0.001, *q* < 0.001) and Tenericutes (rho = 0.45, *p* < 0.001, *q* = 0.001), as was expected due to the differences in these phyla between positive and negative pregnant women (Fig. 3A). However, the results also revealed a negative relation between IgM/A concentration and the Actinobacteria phylum (rho =  − 0.36, *p* = 0.002, q = 0.017) and *Corynebacterium* genus (rho =  − 0.31, *p* = 0.006*, q* = 0.075). At the genus level, IgM/A was positively correlated with *Faecalibacterium* (rho = 0.36, *p* = 0.001*, q* = 0.025), *Subdoligranulum* (rho = 0.43, *p* < 0.001*, q* = 0.003), *Prevotellaceae*_UCG003 (rho = 0.32, *p* = 0.005, q = 0.064) and *Prevotella_1* (rho = 0.42, *p* < 0.001*, q* = 0.004) as well as several groups from the *Ruminococcaceae* family, including the *Ruminococcus_gauvreauii_group* (rho = 0.41, *p* < 0.001*, q* = 0.005) and *Ruminococcus_1* (rho = 0.35, *p* = 0.002*, q* = 0.033) (Fig. 3B). Regarding IgG concentration, a positive association was found with the mentioned genera, including *Prevotella_2* (rho = 0.39, *p* = 0.001*, q* = 0.028), *Prevotellaceae*_UCG003 (rho = 0.33, *p* = 0.003*, q* = 0.110), *Ruminococcus_torques*_group (rho = 0.38, *p* = 0.001*, q* = 0.033) and *Suboligranulum* (rho = 0.44, *p* < 0.001*, q* = 0.007), or uncultured *Porphyromonaceae* family members (rho = 0.5, *p* < 0.001*, q* = 0.001) (Fig. 3B).

### The microbiota composition was similar in COVID-19 women with different clinical severities

Finally, we analyzed the relation with the severity of symptoms; no significant differences were found in terms of alpha diversity among the three severity groups (asymptomatic, mild and severe) (Figure S4); only with the inclusion of negative women was a slightly positive correlation observed between symptom severity numerical variables and both Shannon and observed ASV indexes. Similarly, while the ANCOM test revealed no differences between infected pregnant women with symptoms and those who remained asymptomatic, the Kruskal–Wallis test showed that nasopharyngeal microbiota from asymptomatic women harbored a higher relative abundance of *Enterococcus* (q = 0.004) and *Catenibacterium* (q = 0.014) and a lower proportion of *Ruminococaceae_uncultured* women (q = 0.023).

## Discussion

The present study reports differences in the nasopharyngeal microbial structure and composition of SARS-CoV-2-infected *versus* noninfected pregnant women. SARS-CoV-2-positive pregnant women showed differences in microbiota richness and evenness, with a higher relative abundance of Bacteroidetes (mainly due to the higher abundance of the *Prevotellaceae* family) and Tenericutes phyla. Additionally, we showed that these microbial changes were similar among women with past and present SARS-CoV-2 infection. No significant differences were reported in the most severe cases.

To our knowledge, this study is the first to describe the nasopharyngeal microbiota profile in SARS-CoV-2 infection during pregnancy. Previous studies in nonpregnant COVID-19 individuals reported a similar general microbial composition in the nasopharyngeal tract, with Firmicutes, Bacteroides, Proteobacteria and Actinobacteria as the most relevant phyla^6,9^. In the present study, a different nasopharyngeal profile was reported in pregnancies infected by SARS-CoV-2. Our findings are in agreement with several studies reporting differences in patients with this infection; however, the results are contradictory: Nardelli et al*.*^10^ reported differences in beta diversity, with a reduction in Proteobacteria and Fusobacteria phyla in a subsample of 18 COVID-19 patients. A significant reduction in alpha diversity was reported in 19 COVID-19 patients who were hospitalized in the ICU, whereas no changes were found due to SARS-CoV-2 positivity^7^. In contrast, Ventero et al*.*^6^ did not find any difference in the richness index between positive and negative cases; only in those patients who later developed more severe COVID-19 symptoms was there a loss of network complexity with a higher relative abundance of the *Prevotella* genus. No differences in bacterial richness, diversity or composition between positive and negative SARS-CoV-2 patients were reported by De Maio et al*.*^9^.

Such differences can be related to relatively small sample sizes, different populations and different severities of the condition, which requires several therapies.

Similar findings, such as a higher nasopharyngeal microbiota diversity and richness observed in SARS-CoV-2-infected pregnancies, have also been described in influenza-infected children compared to healthy children^35^. Other studies evaluating other viral infections, mostly influenza virus, have reported that the infection could change the diversity and composition of the nasopharyngeal bacterial community^3,36^. Moreover, in other parts of the respiratory tract, such as lung tissue, several authors reported an enrichment of pathogenic and commensal bacteria in COVID-19 patients^37–40^; in line with this, many authors agree that healthy lung tissue has a low density of microbial populations^41^, and disorders in the microbiota would be characterized by enrichment of OTUs^40^.

It can be hypothesized that respiratory microbial communities could be associated with SARS-CoV-2, without being possible to establish causality: infected patients had an altered respiratory tract microbiome with, in several cases, an increased abundance of OTUs. However, evidence is limited to studies with a relatively small sample size and different participant characteristics. Indeed, nor our study neither some with others with the same study design^6^ was unable to stablish a causal link between SARS-CoV-2 and the alteration in these microbial communities.

This is the first study reporting that changes in the nasopharyngeal microbial community persisted after SARS-CoV-2 infection in pregnant women. This evidence supports the idea of lost-lasting effects of these changes after the acute phase of the infection. Since we did not have a baseline evaluation, we cannot ascertain whether changes in the microbiota were present before the infection; and thus, it is not possible to decipher the role of the SARS-CoV-2 as a potential cause of the observed alterations. However, we believe this is unlikely, considering that other respiratory infections have also been reported to induce changes in the nasopharyngeal microbiota^3,35,36^. An additional finding of this study was the association between taxa overrepresented in SARS-CoV-2-infected women and the levels of IgA/IgM, suggesting a potential relationship between the immune response and the microbiota^42^. Specifically, there was a negative association between IgA/IgM levels and the *Corynebacterium* genus, which is one of the main components of the nasopharyngeal microbiota^43^ and has been related to a healthy condition in several studies^44^ due to its potential capacity to compete with opportunistic pathogens^45^. These findings suggest that the microbiota alterations associated with SARS-CoV-2 could be mediated by the host immune system response.

Other studies have shown how maternal microbiota could change during pregnancy^14^, with a potential role in the initial bacterial seeding of the neonate^46^, which could also contribute to the immune system development during early life^47^. Not only at delivery but also during the gestational process, maternal microbiota has been proposed as a factor to drive fetal development and the susceptibility of the future infant to some diseases^48^. However, nothing is known about how the pregnancy and other related conditions could affect the nasopharyngeal microbiota. Therefore, the present study provided data about a potential association of COVID-19 with microbiota alteration but also provide data about the pregnant women nasopharyngeal microbiota.

In this study, we did not find any differences according to symptom severity. The small subgroup sample size and the high proportion of asymptomatic/mild infections may have hampered observing differences if these existed. Lee et al*.* reported in a nonpregnant population that several species from *Alloprevotella* and *Prevotella* were associated with influenza virus infection^3^. These taxa were also observed to be related to SARS-CoV-2 infection severity by Ventero et al.^6^. Moreover, overexpression of *Prevotella* proteins was related to an increase in the clinical severity of COVID-19^38^. In this study, we found a nonsignificant trend of higher relative abundance of the *Prevotella* genus and several groups from the Ruminococcaceae family.

Our study has some strengths and limitations that deserve comment. Among the strengths, to our knowledge, this is the first study of nasopharyngeal microbiota in pregnant women, providing data about this specific population and opening the door to future studies focused on them. Moreover, nasopharyngeal RT–PCR swab collection was always performed using a standardized procedure from trained medical staff at hospital admission, reducing potential bias before any treatment was started. Moreover, the population was very well characterized by SARS-CoV-2 infection status and COVID-19 symptoms. Nasopharyngeal samples are considered low-biomass samples, and specific positive and negative controls need to be introduced during sequencing to rule out potential contamination and bias due to the low microbial DNA samples. In this study, specific ASV were identified in the negative controls. After filtering process, we further identified specific anaerobic gut microbes, such *Ruminococcus* and *Faecalibacterium*, in the nasopharynx samples, although in lower proportions as other studies also reported to be present in the nasopharynx^49,50^. Furthermore, the butyrate production of those microbial genera would be associated with a reduction in olfactory function^50^, which has been described as a COVID-19 symptom. Another potential explanation would be that the microbial database used as the curation of the open databases is critical for proper identification and reliable taxonomy assignment^51^.

Among limitations, the relatively small sample size did not allow us to draw robust conclusions from subgroup comparisons; additionally, as there were no data on the upper respiratory tract microbiota during pregnancy, it was not possible to discern if changes were due only to the pregnancy status itself and if this could be considered a protective effect for viral infections to become more severe. Finally, future studies are warranted to compare these data with women of the same age but in a nonpregnant status.

## Conclusions

In conclusion, the overall composition and diversity of the nasopharyngeal microbiota differed in pregnant women with and without SARS-CoV-2 infection. These changes were also present in women with a past infection. Further studies are needed to confirm our results and to evaluate the possible clinical implications of nasopharyngeal microbiota alterations in pregnancies complicated with SARS-CoV-2-CoV-2 infection.

## Supplementary Information

Supplementary Information.

## Data Availability

The datasets generated and/or analyzed during the current study are available at this link: https://dataview.ncbi.nlm.nih.gov/object/PRJNA777915?reviewer=8agmagknr66hc31evjrnf7b8ui.
