# Supplementary material for: Nasopharyngeal microbiota profiling of pregnant women with SARS-CoV-2 infection
Source: Sci Rep. 2022 Aug 4;12:13404. doi: 10.1038/s41598-022-17542-z (PMC9352760; doi:10.1038/s41598-022-17542-z)
Supplement: Supplementary file 1 — Supplementary Information. [file 41598_2022_17542_MOESM1_ESM.docx]

**Table S1.** Phylotypes identified as contaminants in the nasopharyngeal microbiota study.

| **Taxa** | **CNP**  **681** | **CNP**  **682** | **CPCR**  **68** | **MOCK**  **68** | **Samples**  **(mean)** |
| --- | --- | --- | --- | --- | --- |
| p__Proteobacteria;c__Alphaproteobacteria;o__Sphingomonadales;f__Sphingomonadaceae;g__Sphingomonas | 6626 | 1369 | 129 | 0 | 1028 |
| p__Firmicutes;c__Erysipelotrichia;o__Erysipelotrichales;f__Erysipelotrichaceae;g__Holdemanella | 0 | 3031 | 0 | 0 | 686.9 |
| p__Actinobacteria;c__Actinobacteria;o__Corynebacteriales;f__Corynebacteriaceae;g__Corynebacterium 1;D_6__Corynebacterium simulans | 0 | 3397 | 0 | 0 | 414.5 |
| p__Actinobacteria;c__Actinobacteria;o__Corynebacteriales;f__Corynebacteriaceae;g__Corynebacterium | 0 | 126 | 0 | 0 | 381.1 |
| p__Actinobacteria;c__Actinobacteria;o__Propionibacteriales;f__Propionibacteriaceae;g__Cutibacterium | 1223 | 0 | 0 | 0 | 379.7 |
| p__Bacteroidetes;c__Bacteroidia;o__Bacteroidales;f__Rikenellaceae;g__Rikenellaceae RC9 gut group;D_6__metagenome | 2569 | 1487 | 0 | 0 | 263.3 |
| p__Firmicutes;c__Erysipelotrichia;o__Erysipelotrichales;f__Erysipelotrichaceae;g__Holdemanella | 0 | 1253 | 0 | 0 | 251.4 |
| p__Firmicutes;c__Clostridia;o__Clostridiales;f__Ruminococcaceae;g__[Eubacterium] coprostanoligenes group;D_6__Clostridiales bacterium 42_27 | 0 | 1690 | 0 | 0 | 224.7 |
| p__Proteobacteria;c__Gammaproteobacteria;o__Pseudomonadales;f__Moraxellaceae;g__Moraxella | 0 | 0 | 301 | 0 | 191.1 |
| p__Firmicutes;c__Negativicutes;o__Selenomonadales;f__Acidaminococcaceae;g__Phascolarctobacterium | 0 | 776 | 0 | 0 | 109.2 |
| p__Actinobacteria;c__Actinobacteria;o__Propionibacteriales;f__Propionibacteriaceae;g__Cutibacterium | 0 | 599 | 0 | 0 | 90.1 |
| p__Firmicutes;c__Clostridia;o__Clostridiales;f__Ruminococcaceae;g__Subdoligranulum | 0 | 519 | 0 | 0 | 80.4 |
| p__Firmicutes;c__Bacilli;o__Lactobacillales;f__Enterococcaceae;g__Enterococcus | 0 | 366 | 0 | 1072 | 57.1 |
| p__Proteobacteria;c__Gammaproteobacteria;o__Enterobacteriales;f__Enterobacteriaceae;g__Escherichia-Shigella | 438 | 0 | 0 | 2155 | 51 |
| p__Proteobacteria;c__Alphaproteobacteria;o__Rhizobiales;f__Beijerinckiaceae;g__Methylobacterium | 0 | 662 | 0 | 0 | 49.2 |
| p__Firmicutes;c__Bacilli;o__Bacillales;f__Bacillaceae;g__Bacillus | 980 | 0 | 0 | 1557 | 46.7 |
| p__Proteobacteria;c__Gammaproteobacteria;o__Betaproteobacteriales;f__Burkholderiaceae;g__Herbaspirillum | 0 | 191 | 0 | 0 | 46.6 |
| p__Bacteroidetes;c__Bacteroidia;o__Bacteroidales;f__Prevotellaceae;g__Prevotellaceae NK3B31 group;D_6__uncultured Prevotella sp. | 0 | 348 | 0 | 0 | 40.4 |
| p__Proteobacteria;c__Gammaproteobacteria;o__Betaproteobacteriales;f__Burkholderiaceae;g__Pelomonas | 371 | 0 | 0 | 0 | 38.3 |
| p__Firmicutes;c__Negativicutes;o__Selenomonadales;f__Veillonellaceae;g__Allisonella;D_6__uncultured bacterium | 0 | 668 | 0 | 0 | 36.7 |
| p__Proteobacteria;c__Gammaproteobacteria;o__Pseudomonadales;f__Moraxellaceae;g__Enhydrobacter | 0 | 850 | 0 | 0 | 35 |
| p__Bacteroidetes;c__Bacteroidia;o__Bacteroidales;f__Muribaculaceae;g__uncultured Porphyromonadaceae bacterium;D_6__uncultured Porphyromonadaceae bacterium | 0 | 297 | 0 | 0 | 34.3 |
| p__Proteobacteria;c__Alphaproteobacteria;o__Rhizobiales;f__Xanthobacteraceae;g__Bradyrhizobium | 0 | 209 | 0 | 0 | 34.2 |
| p__Firmicutes;c__Negativicutes;o__Selenomonadales;f__Acidaminococcaceae;g__Phascolarctobacterium | 0 | 443 | 0 | 0 | 33.6 |
| p__Firmicutes;c__Clostridia;o__Clostridiales;f__Ruminococcaceae;g__Subdoligranulum | 0 | 712 | 0 | 0 | 32.5 |
| p__Firmicutes;c__Negativicutes;o__Selenomonadales;f__Acidaminococcaceae;g__Phascolarctobacterium | 0 | 420 | 0 | 0 | 32.4 |
| p__Firmicutes;c__Clostridia;o__Clostridiales;f__Ruminococcaceae;g__Ruminococcus2 | 0 | 899 | 0 | 0 | 29.1 |
| p__Proteobacteria;c__Gammaproteobacteria;o__Pseudomonadales;f__Moraxellaceae;g__Acinetobacter | 473 | 0 | 0 | 0 | 24 |
| p__Firmicutes;c__Clostridia;o__Clostridiales;f__Ruminococcaceae;g__Ruminococcaceae NK4A214 group | 0 | 514 | 0 | 0 | 23.8 |
| p__Bacteroidetes;c__Bacteroidia;o__Bacteroidales;f__Prevotellaceae;g__Prevotellaceae UCG-003;D_6__uncultured bacterium | 0 | 271 | 0 | 0 | 23.7 |
| p__Proteobacteria;c__Alphaproteobacteria;o__Azospirillales;f__Azospirillaceae;g__Azospirillum | 0 | 343 | 0 | 0 | 23 |
| p__Proteobacteria;c__Gammaproteobacteria;o__Oceanospirillales;f__Halomonadaceae;g__Halomonas | 0 | 212 | 0 | 0 | 22.9 |
| p__Proteobacteria;c__Gammaproteobacteria;o__Xanthomonadales;f__Xanthomonadaceae;g__Stenotrophomonas | 0 | 542 | 0 | 0 | 21.9 |
| p__Firmicutes;c__Bacilli;o__Lactobacillales;f__Lactobacillaceae;g__Lactobacillus | 0 | 135 | 0 | 4088 | 21.4 |
| p__Proteobacteria;c__Gammaproteobacteria;o__Betaproteobacteriales;f__Burkholderiaceae;g__Pelomonas | 0 | 335 | 0 | 0 | 20.7 |
| p__Firmicutes;c__Clostridia;o__Clostridiales;f__Ruminococcaceae;g__[Eubacterium] coprostanoligenes group;D_6__uncultured bacterium | 0 | 149 | 0 | 0 | 16.4 |
| p__Firmicutes;c__Clostridia;o__Clostridiales;f__Ruminococcaceae;g__Ruminococcaceae UCG-002 | 0 | 349 | 0 | 0 | 15.1 |
| p__Bacteroidetes;c__Bacteroidia;o__Bacteroidales;f__Prevotellaceae;g__Prevotella9 | 0 | 415 | 0 | 0 | 11.4 |
| p__Firmicutes;c__Negativicutes;o__Selenomonadales;f__Acidaminococcaceae;g__Phascolarctobacterium | 0 | 346 | 0 | 0 | 11.1 |
| p__Firmicutes;c__Clostridia;o__Clostridiales;f__Lachnospiraceae;g__Agathobacter | 0 | 53 | 0 | 0 | 10.9 |
| p__Firmicutes;c__Clostridia;o__Clostridiales;f__FamilyXIII;g__FamilyXIII AD3011 group;D_6__uncultured Eubacterium sp. | 0 | 118 | 0 | 0 | 10.2 |
| p__Bacteroidetes;c__Bacteroidia;o__Bacteroidales;f__Rikenellaceae;g__Rikenellaceae RC9 gut group;D_6__uncultured bacterium | 0 | 30 | 0 | 0 | 8.3 |
| p__Proteobacteria;c__Gammaproteobacteria;o__Betaproteobacteriales;f__Burkholderiaceae;g__Curvibacter | 0 | 88 | 0 | 0 | 6.7 |

Columns present the number of the reads classified as the specific phylotype in the negative controls (CNP681 and CNP682: controls during DNA extraction; CPCR68: control library preparation; MOCK68: positive control mock community for sequencing) and the mean of reads detected in all the samples. Phylotype are identified as its taxonomic classification genus level (species level when the identification was available).

**Table S2.** Baseline characteristics of the study population subdivided according to SARS-CoV-2 infection.

|  | **SARS-CoV-2 negative** | **SARS-CoV-2 positive** | **P** | |
| --- | --- | --- | --- | --- |
|  | (n=38) | (n=38) |  | |
| Age, years | 31.3 (6.2) | 31.4 (5.8) | 0.948 | |
| Race or ethnic group |  |  |  | |
| White | 24 (63.2%) | 24 (63.2%) | 0.594 | |
| Latin-American | 9 (23.7%) | 6 (15.8%) | 0.283 | |
| Asian | 3 (7.9%) | 5 (13.2%) | 0.356 | |
| Others | 2 (5.3%) | 3 (7.9%) | 0.5 | |
| Low socioeconomical status^#^ | 13 (34.2%) | 12 (31.6%) | 0.5 | |
| Body mass index, kg/m^2^ | 24.2 (5) | 24.7 (4.3) | 0.598 | |
| Smoking during pregnancy | 6 (15.8%) | 2 (5.3%) | 0.131 | |
| Chronic hypertension | 1 (2.6%) | 1 (2.6%) | 0.753 | |
| Diabetes mellitus | 1 (2.6%) | 1 (2.6%) | 0.753 | |
| Obesity^†^ | 6 (15.8%) | 4 (10.5%) | 0.368 | |
| Asthma | 4 (10.5%) | 6 (15.8%) | 0.368 |  |
| Hypothyroidism | 5 (13.2%) | 5 (13.2%) | 0.632 | |
| Pregnancy history |  |  |  | |
| Nulliparous | 17 (44.7%) | 16 (42.1%) | 0.5 | |
| Assisted reproductive technologies | 2 (5.3%) | 4 (10.5%) | 0.337 | |
| Multiple gestation | 1 (2.6%) | 0 (0%) | 0.5 | |
| Gestational age at recruitment, weeks | 39.6 (2.3) | 39.4 (1.8) | 0.701 | |

Data are n (%) or or mean ± SD.

^#^Low socioeconomic status defined as no studies, never worked or unemployment for two years or more

^†^Obesity defined as body mass index >30 kg/m^2^.

SARS-CoV-2: severe acute respiratory syndrome coronavirus 2 (SARS-CoV-2)

**Table S3.** Pregnancy and perinatal outcomes in women with and without evidence of SARS-CoV-2 infection

|  | **SARS-CoV-2**  **negative**  (n=38) | | **SARS-CoV-2 positive**  (n=38) | **p** |
| --- | --- | --- | --- | --- |
| *Pregnancy outcomes* | | | | |
| Iron intake | | 23 (60.5%) | 13 (34.2%) | 0.022 |
| Antibiotics during pregnancy | | 3 (7.9%) | 4 (10.5%) | 0.692 |
| Preeclampsia | | 4 (10.5%) | 0 (0%) | 0.040 |
| Gestational diabetes mellitus | | 3 (7.9%) | 3 (7.9%) | 1.000 |
| Spontaneous preterm labour | | 2 (5.3%) | 2 (5.3%) | 1.000 |
| Maternal hospital stay, days | | 2 (2-3) | 2 (2-3) | 0.894 |
| *Delivery outcomes* | | | | |
| Induction of labour | | 18 (47.4%) | 15 (39.5%) | 0.488 |
| Caesarean section | | 8 (21.1%) | 14 (36.8%) | 0.129 |
| Antibiotics during delivery | | 14 (36.8) | 14 (36.8) | 1.000 |
| Gestational age at delivery, weeks | | 39.5 ± 2.2 | 39.5 ± 1.3 | 0.965 |
| Preterm labour | | 2 (5.3%) | 2 (5.3%) | 1.000 |
| Birth weight, g | | 3287 ± 575 | 3203 ± 483 | 0.493 |
| Birth weight percentile | | 46 (26-71) | 39 (13-69) | 0.335 |
| Small-for-gestational age^#^ | | 5 (13.2%) | 6 (15.8%) | 0.744 |
| Admission to neonatal intensive care unit | | 4 (10.5%) | 0 (0%) | 0.040 |
| Maternal breastfeeding | | 35 (92.1%) | 36 (94.7%) | 0.644 |

Data are n (%) or median (IQR) or mean ± SD.

^#^Small-for-gestational age defined as birth weight below the 10^th^ centile.

SARS-CoV-2: severe acute respiratory syndrome coronavirus 2 (SARS-CoV-2).

**TTable S4.** Differences in the nasopharyngeal microbiota at phylum level according to SARS-CoV-2 infection determined by ANCOM test

| **Phylum** | **SARS-COV-2 Negative** | **SARS-COV-2 Positive** | **clr** | **W** |
| --- | --- | --- | --- | --- |
| Firmicutes | 8707 [5278.3- 11625] | 8891.5 [7001- 13646.3] | 0.10 | 3 |
| Proteobacteria | 6915.5 [4428.8- 9807.8] | 6299 [3851.5- 8462] | 0.36 | 2 |
| Actinobacteria | 6662.5 [3807.8- 9557.8] | 4716 [2387.8- 5631.8] | 0.60 | 3 |
| **Bacteroidetes** | **948 [287.8- 2963.8]** | **4795.5 [3109.5- 7729.5]** | **-1.95** | **29*** |
| Cyanobacteria | 1 [1- 436.8] | 266 [1- 581.3] | -0.86 | 0 |
| **Tenericutes** | **1 [1- 1]** | **109 [10.3- 416.8]** | **-2.61** | **31*** |
| Fusobacteria | 1 [1- 1] | 1 [1- 431.8] | -1.27 | 0 |
| Patescibacteria | 1 [1- 104.8] | 1 [1- 229.5] | 0.08 | 1 |
| Epsilonbacteraeota | 1 [1- 1] | 1 [1- 167.8] | -0.40 | 0 |
| Acidobacteria | 1 [1- 1] | 1 [1- 1] | 0.49 | 2 |
| Armatimonadetes | 1 [1- 1] | 1 [1- 1] | 0.19 | 2 |
| Chlamydiae | 1 [1- 1] | 1 [1- 1] | 0.15 | 2 |
| Chloroflexi | 1 [1- 1] | 1 [1- 1] | 0.15 | 2 |
| Deinococcus-Thermus | 1 [1- 1] | 1 [1- 1] | -0.25 | 1 |
| Dependentiae | 1 [1- 1] | 1 [1- 1] | 0.31 | 2 |
| FBP | 1 [1- 1] | 1 [1- 1] | 0.05 | 2 |
| Fibrobacteres | 1 [1- 1] | 1 [1- 1] | 0.25 | 2 |
| Gemmatimonadetes | 1 [1- 1] | 1 [1- 1] | 0.13 | 2 |
| Halanaerobiaeota | 1 [1- 1] | 1 [1- 1] | 0.34 | 2 |
| Kiritimatiellaeota | 1 [1- 1] | 1 [1- 1] | 0.48 | 2 |
| Latescibacteria | 1 [1- 1] | 1 [1- 1] | 0.38 | 2 |
| Margulisbacteria | 1 [1- 1] | 1 [1- 1] | 0.12 | 2 |
| Nitrospirae | 1 [1- 1] | 1 [1- 1] | 0.39 | 2 |
| Planctomycetes | 1 [1- 1] | 1 [1- 1] | 0.58 | 2 |
| Spirochaetes | 1 [1- 1] | 1 [1- 1] | -0.10 | 1 |
| Synergistetes | 1 [1- 1] | 1 [1- 1] | -0.05 | 2 |
| TA06 | 1 [1- 1] | 1 [1- 1] | 0.30 | 2 |
| Verrucomicrobia | 1 [1- 1] | 1 [1- 1] | 0.78 | 2 |
| Zixibacteria | 1 [1- 1] | 1 [1- 1] | 0.31 | 2 |
| Unclassified | 1 [1- 1] | 1 [1- 1] | 0.33 | 2 |

Columns NEG and POS present the median and the interquartile range (median [IQR]) of the reads classified as the specific taxa in both categories, respectively. Taxa with significant differences assessed by ANCOM test are marked in bold.

**Table S5.** Differences in the nasopharyngeal microbiota at genus level according to SARS-CoV-2 infection determined by ANCOM test

| **Taxa** | **SARS-CoV-2 Negative** | **SARS-CoV-2 Positive** | **clr** | **W** |
| --- | --- | --- | --- | --- |
| k_Bacteria;p_Firmicutes;c_Clostridia;o_Clostridiales;f_Family XI;g_Peptoniphilus | 91 [1-855.3] | 1 [1-226] | -1.12 | 19 |
| k_Bacteria;p_Proteobacteria;c_Alphaproteobacteria;o_Azospirillales;f_Azospirillaceae;g_Azospirillum | 8 [1-170.3] | 126 [1-169.8] | 0.63 | 4 |
| k_Bacteria;p_Actinobacteria;c_Actinobacteria;o_Corynebacteriales;f_Corynebacteriaceae;g_Lawsonella | 68 [1-541.5] | 98.5 [1-365.5] | -0.53 | 11 |
| k_Bacteria;p_Proteobacteria;c_Alphaproteobacteria;o_Rhizobiales;f_Beijerinckiaceae;g_Methylobacterium | 62 [1-284.5] | 65 [14-229] | 0.00 | 15 |
| k_Bacteria;p_Proteobacteria;c_Gammaproteobacteria;o_Oceanospirillales;f_Halomonadaceae;g_Halomonas | 60 [7.3-328.75] | 103 [28.5-297.8] | 0.00 | 13 |
| k_Bacteria;p_Firmicutes;c_Bacilli;o_Lactobacillales;f_Enterococcaceae;g_Enterococcus | 57.5 [1-172] | 99.5 [19-157.8] | 0.62 | 6 |
| k_Bacteria;p_Firmicutes;c_Clostridia;o_Clostridiales;f_Family XI;g_Anaerococcus | 53 [1-734] | 63.5 [1-324.8] | -0.54 | 12 |
| k_Bacteria;p_Proteobacteria;c_Gammaproteobacteria;o_Betaproteobacteriales;f_Burkholderiaceae;g_Massilia | 43.5 [1-164.3] | 73 [31.5-232] | 0.42 | 10 |
| k_Bacteria;p_Actinobacteria;c_Actinobacteria;o_Propionibacteriales;f_Propionibacteriaceae;g_Cutibacterium | 425 [133-776] | 528.5 [298.8-855.3] | 0.20 | 17 |
| k_Bacteria;p_Proteobacteria;c_Gammaproteobacteria;o_Pseudomonadales;f_Pseudomonadaceae;g_Pseudomonas | 412.5 [23.5-938.5] | 390 [230.8-717.8] | 1.05 | 4 |
| k_Bacteria;p_Actinobacteria;c_Actinobacteria;o_Corynebacteriales;f_Corynebacteriaceae;g_Corynebacterium 1 | 4004 [1560.5-7077.5] | 2310 [1185.3-3777] | -0.56 | 20 |
| k_Bacteria;p_Proteobacteria;c_Gammaproteobacteria;o_Enterobacteriales;f_Enterobacteriaceae;g_Escherichia-Shigella | 29.5 [1-92] | 7.5 [1-110] | -0.56 | 19 |
| k_Bacteria;p_Firmicutes;c_Bacilli;o_Lactobacillales;f_Streptococcaceae;g_Streptococcus | 276 [8-716.75] | 399 [57-833.8] | 0.35 | 4 |
| k_Bacteria;p_Proteobacteria;c_Gammaproteobacteria;o_Betaproteobacteriales;f_Burkholderiaceae;g_Pelomonas | 18 [1-83.3] | 104 [1-273.3] | 1.03 | 4 |
| k_Bacteria;p_Proteobacteria;c_Gammaproteobacteria;o_Betaproteobacteriales;f_Neisseriaceae;g_uncultured | 178.5 [1-1082.8] | 134.5 [1-910.8] | -0.66 | 10 |
| k_Bacteria;p_Proteobacteria;c_Alphaproteobacteria;o_Rhizobiales;f_Xanthobacteraceae;g_Bradyrhizobium | 161 [38-331.5] | 239 [77.8-408] | 0.32 | 12 |
| k_Bacteria;p_Firmicutes;c_Bacilli;o_Bacillales;f_Staphylococcaceae;g_Staphylococcus | 1594 [625.3-4686.3] | 1633 [639.5-2668] | -0.02 | 14 |
| k_Bacteria;p_Proteobacteria;c_Gammaproteobacteria;o_Pseudomonadales;f_Moraxellaceae;g_Acinetobacter | 139.5 [9.3-273.3] | 115 [9.8-261.5] | -0.21 | 13 |
| k_Bacteria;p_Proteobacteria;c_Alphaproteobacteria;o_Sphingomonadales;f_Sphingomonadaceae;g_Sphingomonas | 1148 [401.3-2147.8] | 1449.5 [1040.8-1815.8] | 0.38 | 16 |
| k_Bacteria;p_Firmicutes;c_Clostridia;o_Clostridiales;f_Ruminococcaceae;g_[Eubacterium] coprostanoligenes group | 10.5 [1-125.3] | 420.5 [180.5-929.3] | 3.14 | 724 |
| k_Bacteria;p_Proteobacteria;c_Alphaproteobacteria;o_Sphingomonadales;f_Sphingomonadaceae;g_Sphingobium | 1 [1-96.5] | 51 [1-197] | 0.37 | 6 |
| k_Bacteria;p_Firmicutes;c_Clostridia;o_Clostridiales;f_Ruminococcaceae;g_Ruminococcaceae UCG-014 | 1 [1-94.5] | 205.5 [107.5-330.8] | 2.74 | 686 |
| k_Bacteria;p_Firmicutes;c_Bacilli;o_Bacillales;f_Bacillaceae;g_Anaerobacillus | 1 [1-80.5] | 13 [1-92] | 0.07 | 8 |
| k_Bacteria;p_Zixibacteria;c_uncultured bacterium;o_uncultured bacterium;f_uncultured bacterium;g_uncultured | 1 [1-8] | 1 [1-1] | -0.22 | 21 |
| k_Bacteria;p_Proteobacteria;c_Gammaproteobacteria;o_Betaproteobacteriales;f_Burkholderiaceae;g_Herbaspirillum | 1 [1-74.8] | 57 [1-189.5] | 0.69 | 4 |
| k_Bacteria;p_Proteobacteria;c_Gammaproteobacteria;o_Pasteurellales;f_Pasteurellaceae;g_Haemophilus | 1 [1-71.5] | 1 [1-253.8] | 0.54 | 5 |
| k_Bacteria;p_Actinobacteria;c_Actinobacteria;o_Actinomycetales;f_Actinomycetaceae;g_Actinomyces | 1 [1-7] | 1 [1-40] | 0.01 | 12 |
| k_Bacteria;p_Firmicutes;c_Clostridia;o_Clostridiales;f_Ruminococcaceae;g_Faecalibacterium | 1 [1-49.8] | 320 [165.8-555.5] | 3.49 | 748 |
| k_Bacteria;p_Proteobacteria;c_Alphaproteobacteria;o_Rhodobacterales;f_Rhodobacteraceae;g_Paracoccus | 1 [1-48.5] | 1 [1-117.3] | 0.31 | 8 |
| k_Bacteria;p_Cyanobacteria;c_Oxyphotobacteria;o_Chloroplast;__;__ | 1 [1-43] | 1 [1-320.5] | 0.79 | 2 |
| k_Bacteria;p_Firmicutes;c_Clostridia;o_Clostridiales;f_Ruminococcaceae;g_Ruminococcaceae UCG-002 | 1 [1-40.3] | 78 [3-232.8] | 1.53 | 1 |
| k_Bacteria;p_Firmicutes;c_Negativicutes;o_Selenomonadales;f_Veillonellaceae;g_Veillonella | 1 [1-4.8] | 1 [1-208.8] | 1.04 | 1 |
| k_Bacteria;p_Firmicutes;c_Erysipelotrichia;o_Erysipelotrichales;f_Erysipelotrichaceae;g_Catenibacterium | 1 [1-30.3] | 1 [1-49.8] | -0.11 | 13 |
| k_Bacteria;p_Proteobacteria;c_Gammaproteobacteria;o_Enterobacteriales;f_Enterobacteriaceae;g_Serratia | 1 [1-30.3] | 1 [1-64] | 0.05 | 12 |
| k_Bacteria;p_Firmicutes;c_Bacilli;o_Lactobacillales;f_Lactobacillaceae;g_Lactobacillus | 1 [1-3.3] | 46 [1-163.3] | 1.42 | 4 |
| k_Bacteria;p_Proteobacteria;c_Gammaproteobacteria;o_Betaproteobacteriales;f_Neisseriaceae;g_Neisseria | 1 [1-27.3] | 1 [1-74.8] | 0.02 | 7 |
| k_Bacteria;p_Firmicutes;c_Clostridia;o_Clostridiales;f_Ruminococcaceae;g_Ruminococcaceae NK4A214 group | 1 [1-23.3] | 49.5 [1-127.3] | 1.25 | 5 |
| k_Bacteria;Unclassified | 1 [1-21] | 1 [1-17] | -0.25 | 21 |
| k_Bacteria;p_Bacteroidetes;c_Bacteroidia;o_Bacteroidales;f_Prevotellaceae;g_Alloprevotella | 1 [1-21.3] | 17 [1-273.8] | 1.03 | 1 |
| k_Bacteria;p_Firmicutes;c_Erysipelotrichia;o_Erysipelotrichales;f_Erysipelotrichaceae;g_Solobacterium | 1 [1-17] | 1 [1-69] | 0.59 | 7 |
| k_Bacteria;p_Bacteroidetes;c_Bacteroidia;o_Bacteroidales;f_Prevotellaceae;g_Prevotella 1 | 1 [1-168.5] | 682 [282-1075.5] | 3.42 | 729 |
| k_Bacteria;p_Firmicutes;c_Bacilli;o_Lactobacillales;f_Carnobacteriaceae;g_Dolosigranulum | 1 [1-1377.8] | 1 [1-1008.5] | -0.01 | 3 |
| k_Bacteria;p_Bacteroidetes;c_Bacteroidia;o_Bacteroidales;f_Prevotellaceae;g_Prevotella 7 | 1 [1-132] | 1 [1-154.5] | -0.19 | 9 |
| k_Bacteria;p_Firmicutes;c_Bacilli;o_Bacillales;f_Bacillaceae;g_Bacillus | 1 [1-126] | 36 [1-118] | 0.10 | 13 |
| k_Bacteria;p_Bacteroidetes;c_Bacteroidia;o_Bacteroidales;f_Rikenellaceae;g_Rikenellaceae RC9 gut group | 1 [1-122.5] | 377 [191.5-661.5] | 3.08 | 714 |
| k_Bacteria;p_Bacteroidetes;c_Bacteroidia;o_Bacteroidales;f_Prevotellaceae;g_Prevotella | 1 [1-121] | 1 [1-128.5] | -0.18 | 8 |
| k_Bacteria;p_Firmicutes;c_Erysipelotrichia;o_Erysipelotrichales;f_Erysipelotrichaceae;g_Erysipelothrix | 1 [1-110] | 38 [1-104.5] | 0.17 | 9 |
| k_Bacteria;p_Proteobacteria;c_Gammaproteobacteria;o_Pseudomonadales;f_Moraxellaceae;g_Moraxella | 1 [1-1015] | 1 [1-72.5] | -0.73 | 14 |
| k_Bacteria;p_Proteobacteria;c_Gammaproteobacteria;o_Enterobacteriales;f_Enterobacteriaceae;__ | 1 [1-10.8] | 1 [1-1] | -0.60 | 20 |
| k_Bacteria;p_Bacteroidetes;c_Bacteroidia;o_Bacteroidales;f_Muribaculaceae;g_uncultured Porphyromonadaceae | 1 [1-1] | 477.5 [218.3-914] | 4.43 | 757 |
| k_Bacteria;p_Bacteroidetes;c_Bacteroidia;o_Bacteroidales;f_Tannerellaceae;g_Parabacteroides | 1 [1-1] | 368 [107.5-624.8] | 4.41 | 757 |
| k_Bacteria;p_Firmicutes;c_Clostridia;o_Clostridiales;f_Lachnospiraceae;g_[Ruminococcus] torques group | 1 [1-1] | 402 [160.3-1049.8] | 4.34 | 757 |
| k_Bacteria;p_Bacteroidetes;c_Bacteroidia;o_Bacteroidales;f_Muribaculaceae;g_uncultured bacterium | 1 [1-1] | 354.5 [136.5-590.5] | 4.01 | 756 |
| k_Bacteria;p_Firmicutes;c_Negativicutes;o_Selenomonadales;f_Acidaminococcaceae;g_Phascolarctobacterium | 1 [1-1] | 391 [92.5-929.5] | 4.02 | 756 |
| k_Bacteria;p_Bacteroidetes;c_Bacteroidia;o_Bacteroidales;f_Prevotellaceae;g_Prevotellaceae NK3B31 group | 1 [1-1] | 296.5 [27-469.5] | 3.60 | 751 |
| k_Bacteria;p_Actinobacteria;c_Coriobacteriia;o_Coriobacteriales;f_Coriobacteriaceae;g_Collinsella | 1 [1-1] | 151.5 [67.3-302.5] | 3.42 | 750 |
| k_Bacteria;p_Firmicutes;c_Negativicutes;o_Selenomonadales;f_Veillonellaceae;g_Allisonella | 1 [1-1] | 156 [68.5-321] | 3.41 | 748 |
| k_Bacteria;p_Firmicutes;c_Clostridia;o_Clostridiales;f_Ruminococcaceae;g_Subdoligranulum | 1 [1-1] | 132.5 [64.3-235.3] | 3.38 | 747 |
| k_Bacteria;p_Bacteroidetes;c_Bacteroidia;o_Bacteroidales;f_Prevotellaceae;__ | 1 [1-1] | 335 [30.3-601.8] | 3.43 | 746 |
| k_Bacteria;p_Firmicutes;c_Clostridia;o_Clostridiales;f_Ruminococcaceae;g_Ruminococcus 2 | 1 [1-1] | 225 [41.3-442.3] | 3.34 | 743 |
| k_Bacteria;p_Firmicutes;c_Clostridia;o_Clostridiales;f_Lachnospiraceae;__ | 1 [1-1] | 130.5 [42.3-273.8] | 2.89 | 715 |
| k_Bacteria;p_Bacteroidetes;c_Bacteroidia;o_Bacteroidales;f_Prevotellaceae;g_Prevotella 9 | 1 [1-1] | 282.5 [1-919.8] | 3.02 | 698 |
| k_Bacteria;p_Firmicutes;c_Clostridia;o_Clostridiales;f_Family XIII;g_Family XIII AD3011 group | 1 [1-1] | 71 [1-174.3] | 2.53 | 675 |
| k_Bacteria;p_Firmicutes;c_Erysipelotrichia;o_Erysipelotrichales;f_Erysipelotrichaceae;g_Holdemanella | 1 [1-1] | 138 [10.5-333.5] | 2.77 | 674 |
| k_Bacteria;p_Firmicutes;c_Clostridia;o_Clostridiales;f_Ruminococcaceae;__ | 1 [1-1] | 32 [1-78.3] | 1.73 | 431 |
| k_Bacteria;p_Firmicutes;c_Negativicutes;o_Selenomonadales;f_Veillonellaceae;g_Anaerovibrio | 1 [1-1] | 1 [1-18.3] | 0.88 | 12 |
| k_Bacteria;p_Firmicutes;c_Negativicutes;o_Selenomonadales;f_Veillonellaceae;g_Megasphaera | 1 [1-1] | 1 [1-49.3] | 0.57 | 12 |
| k_Bacteria;p_Firmicutes;c_Clostridia;o_Clostridiales;f_Peptostreptococcaceae;__ | 1 [1-1] | 1 [1-29.3] | 0.90 | 11 |
| k_Bacteria;p_Firmicutes;c_Clostridia;o_Clostridiales;f_Ruminococcaceae;g_Ruminococcaceae UCG-010 | 1 [1-1] | 1 [1-16.8] | 0.61 | 11 |
| k_Bacteria;p_Firmicutes;c_Clostridia;o_Clostridiales;f_Lachnospiraceae;g_Lachnospiraceae FCS020 group | 1 [1-1] | 1 [1-67.3] | 1.33 | 10 |
| k_Bacteria;p_Firmicutes;c_Clostridia;o_Clostridiales;__;__ | 1 [1-1] | 1 [1-37] | 1.09 | 9 |
| k_Bacteria;p_Proteobacteria;c_Alphaproteobacteria;o_Rickettsiales;f_Mitochondria;__ | 1 [1-1] | 1 [1-13] | 0.61 | 9 |
| k_Bacteria;p_Bacteroidetes;c_Bacteroidia;o_Bacteroidales;f_Prevotellaceae;g_uncultured | 1 [1-1] | 1 [1-61] | 0.82 | 8 |
| k_Bacteria;p_Firmicutes;c_Clostridia;o_Clostridiales;f_Ruminococcaceae;g_Ruminococcaceae UCG-005 | 1 [1-1] | 1 [1-32.5] | 0.84 | 7 |
| k_Bacteria;p_Tenericutes;c_Mollicutes;o_Mollicutes RF39;f_uncultured bacterium;g_uncultured bacterium | 1 [1-1] | 1 [1-31.5] | 1.10 | 7 |
| k_Bacteria;p_Proteobacteria;c_Alphaproteobacteria;o_Caulobacterales;f_Caulobacteraceae;g_Brevundimonas | 1 [1-1] | 1 [1-85] | 0.81 | 6 |
| k_Bacteria;p_Proteobacteria;c_Gammaproteobacteria;o_Betaproteobacteriales;f_Burkholderiaceae;g_Aquabacterium | 1 [1-1] | 1 [1-43.8] | 0.61 | 6 |
| k_Bacteria;p_Proteobacteria;c_Gammaproteobacteria;o_Betaproteobacteriales;f_Burkholderiaceae;g_Curvibacter | 1 [1-1] | 1 [1-52.5] | 0.83 | 6 |
| k_Bacteria;p_Tenericutes;c_Mollicutes;o_Mollicutes RF39;__;__ | 1 [1-1] | 1 [1-59] | 1.24 | 6 |
| k_Bacteria;p_Epsilonbacteraeota;c_Campylobacteria;o_Campylobacterales;f_Campylobacteraceae;g_Campylobacter | 1 [1-1] | 1 [1-167.8] | 0.48 | 5 |
| k_Bacteria;p_Proteobacteria;c_Gammaproteobacteria;o_Betaproteobacteriales;f_Burkholderiaceae;g_Ralstonia | 1 [1-1] | 1 [1-55.8] | 0.72 | 5 |
| k_Bacteria;p_Bacteroidetes;c_Bacteroidia;o_Bacteroidales;f_Porphyromonadaceae;g_Porphyromonas | 1 [1-1] | 1 [1-109] | 0.39 | 4 |
| k_Bacteria;p_Proteobacteria;c_Gammaproteobacteria;o_Pseudomonadales;f_Moraxellaceae;g_Enhydrobacter | 1 [1-1] | 1 [1-66.5] | 0.72 | 4 |
| k_Bacteria;p_Tenericutes;c_Mollicutes;o_Mollicutes RF39;f_gut metagenome;g_gut metagenome | 1 [1-1] | 1 [1-96.5] | 1.45 | 4 |
| k_Bacteria;p_Firmicutes;c_Clostridia;o_Clostridiales;f_Lachnospiraceae;g_Coprococcus 3 | 1 [1-1] | 36.5 [1-131.8] | 1.45 | 3 |
| k_Bacteria;p_Fusobacteria;c_Fusobacteriia;o_Fusobacteriales;f_Fusobacteriaceae;g_Fusobacterium | 1 [1-1] | 1 [1-151.8] | 0.50 | 3 |
| k_Bacteria;p_Fusobacteria;c_Fusobacteriia;o_Fusobacteriales;f_Leptotrichiaceae;g_Leptotrichia | 1 [1-1] | 1 [1-176.8] | 1.08 | 3 |
| k_Bacteria;p_Proteobacteria;c_Gammaproteobacteria;o_Xanthomonadales;f_Xanthomonadaceae;g_Stenotrophomonas | 1 [1-1] | 1 [1-128.8] | 1.01 | 3 |
| k_Bacteria;p_Firmicutes;c_Clostridia;o_Clostridiales;f_Lachnospiraceae;g_Agathobacter | 1 [1-1] | 1 [1-80.3] | 1.52 | 16 |
| k_Bacteria;p_Firmicutes;c_Erysipelotrichia;o_Erysipelotrichales;f_Erysipelotrichaceae;g_uncultured | 1 [1-1] | 1 [1-42.3] | 1.25 | 16 |
| k_Bacteria;p_Firmicutes;c_Negativicutes;o_Selenomonadales;f_Veillonellaceae;g_Selenomonas | 1 [1-1] | 1 [1-18.3] | 0.64 | 15 |
| k_Bacteria;p_Actinobacteria;c_Coriobacteriia;o_Coriobacteriales;f_Atopobiaceae;g_uncultured | 1 [1-1] | 1 [1-13.3] | 0.82 | 13 |
| k_Bacteria;p_Actinobacteria;c_Coriobacteriia;o_Coriobacteriales;f_Eggerthellaceae;g_uncultured | 1 [1-1] | 1 [1-8.5] | 0.69 | 13 |

Only those genera with an interquartile range>1 for at least one of the studied categories (Positive and Negative) are shown. Columns Positive and Negative present the median and the interquartile range (median [IQR]) of the reads classified as the specific taxa in both categories, respectively. Taxa with significant differences assessed by ANCOM test are marked in bold.

**SUPPLEMENTARY FIGURES**

A)


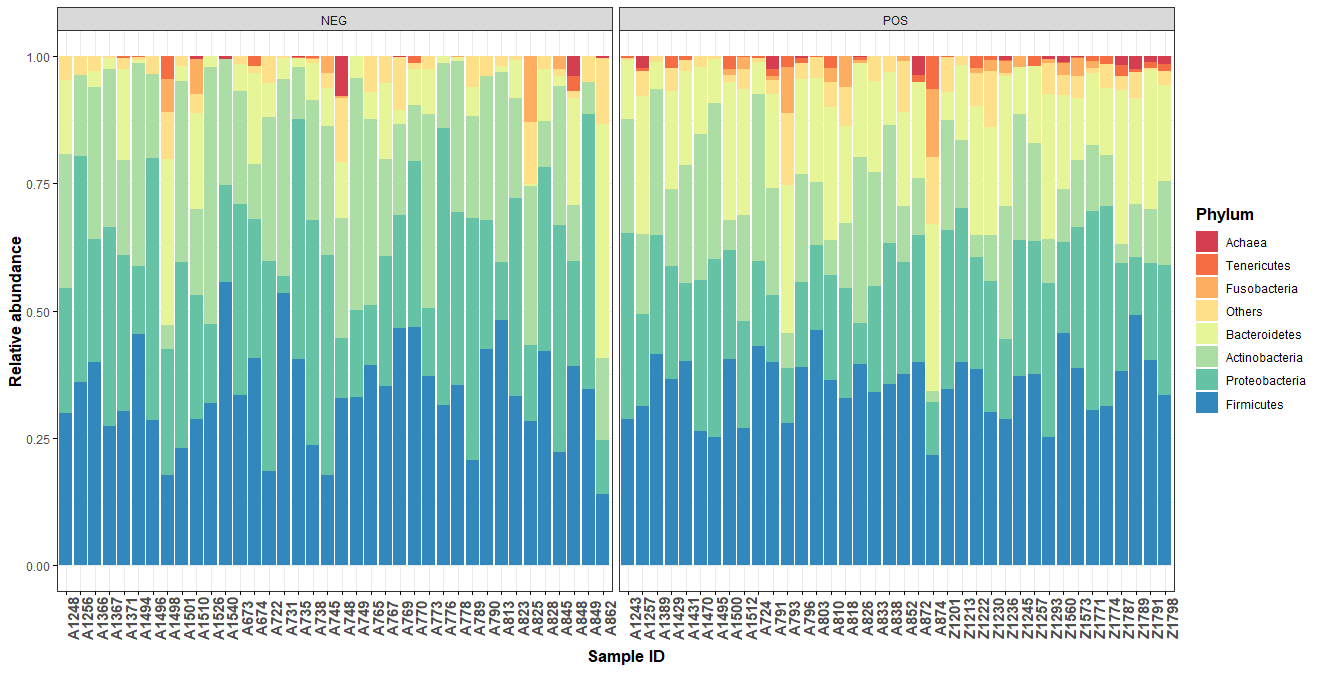


B)


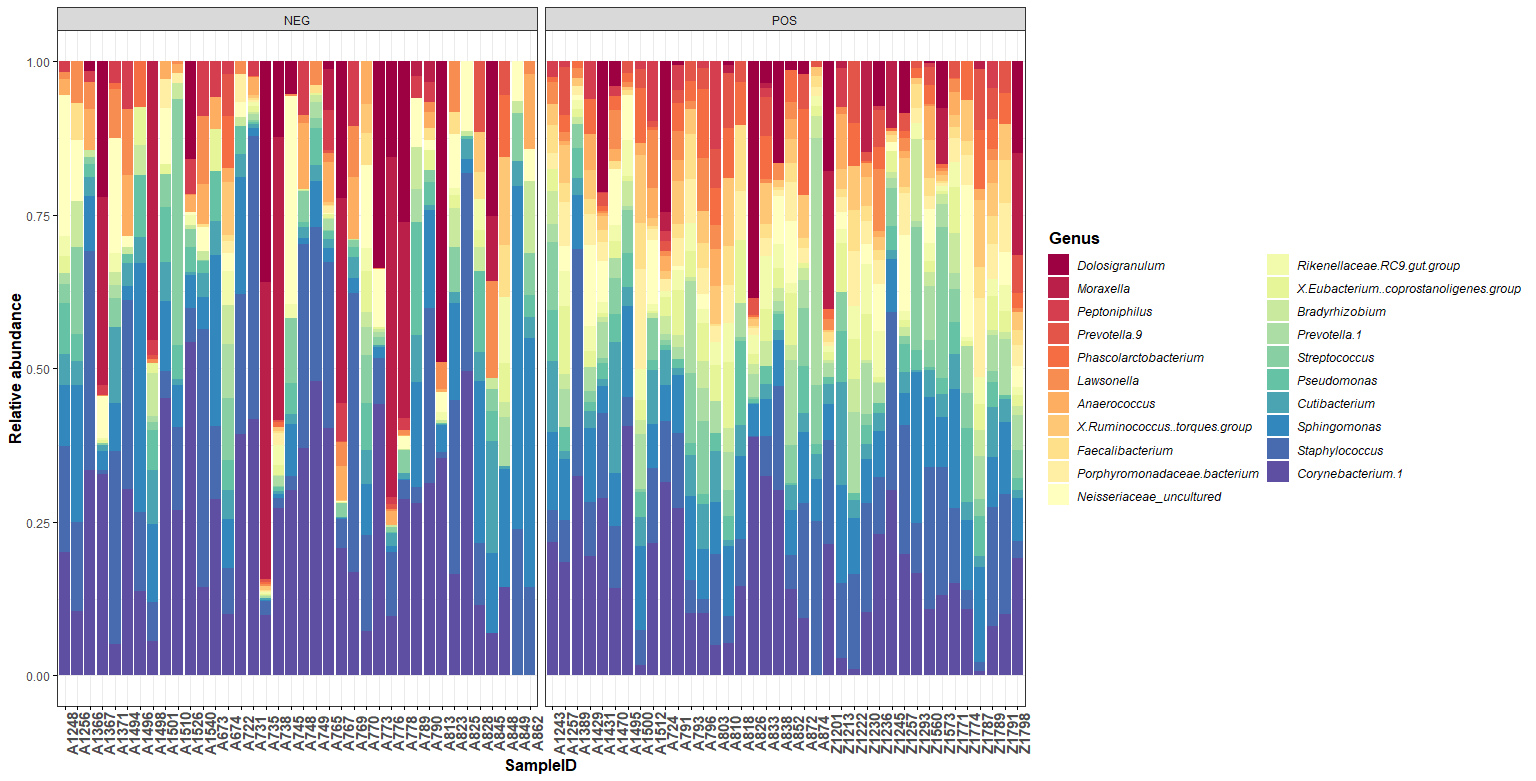


**Figure S1.** Description of nasopharyngeal microbiota at phylum (A) and genus level (B) from the studied population classified according their result for SARS-CoV-2 infection. At phylum level, those phyla with a relative abundance lower than 0.5% and Cyanobacteria were grouped as “Others”. Only those genera with a relative abundance higher than 0.1% are shown in figure. POS: Positive SARS-CoV-2 infection, NEG: Negative SARS-CoV-2 infection.

A)


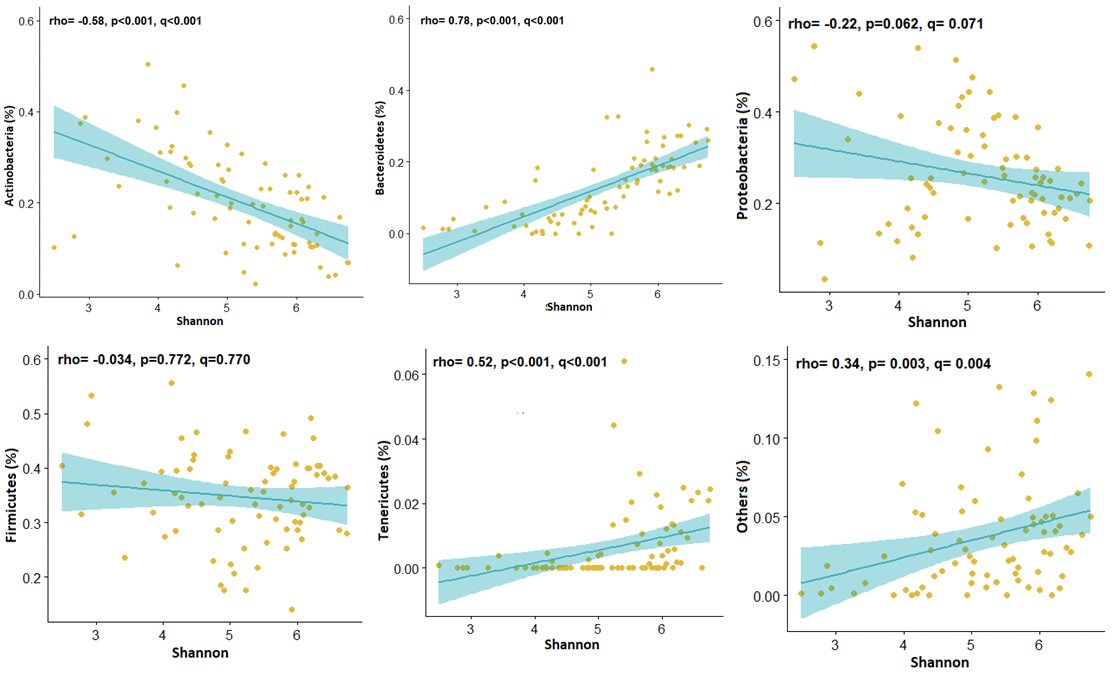


B)


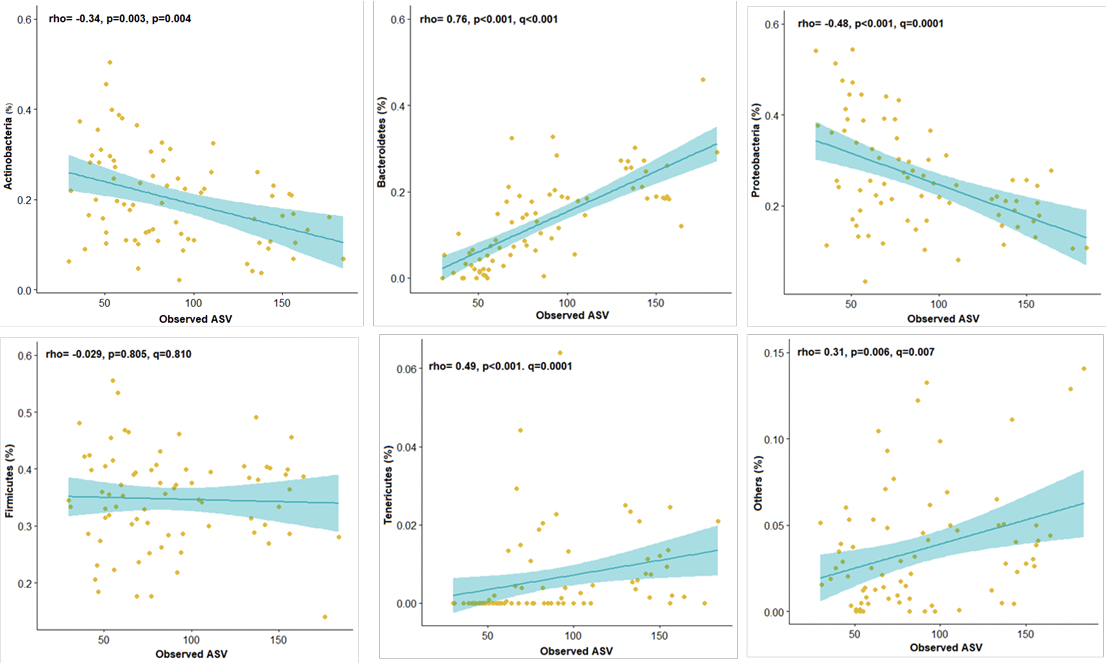


**Figure S2.** Scatter plots of the Spearman correlations between the relative abundance of the nasopharingeal microbial phyla and both alpha-diversity index, Shannon index (A) and Observed ASV (B). One outlayer point was removed for ploting (Observed ASV) after testing that the significant correlations were not affected by this individual.


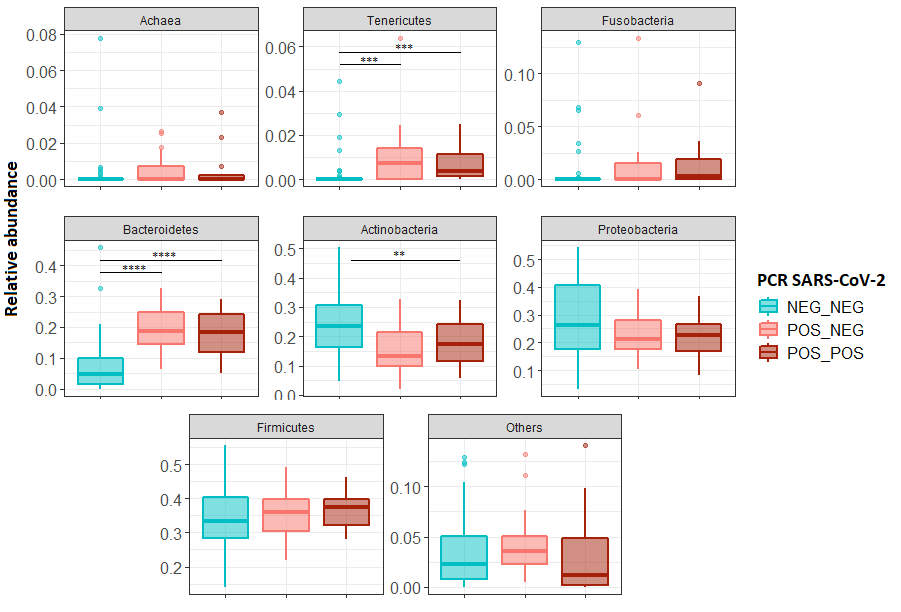


**Figure S3.** Differences in nasopharyngeal microbial phyla between those mothers with positive and negative (positive by serological determination) result for SARS-CoV-2 infection. At phylum level, those phyla with a relative abundance lower tan 0.5 and Cyanobacteria were grouped as “Others” but considered individually for all statistical analysis. **POS_POS**: Positive result for the serological determination and also for RT-PCR of SARS-CoV-2 (active infection); **POS_NEG**: Positive result in the serological determination and negative for for RT-PCR of SARS-CoV-2 (past infection); **NEG-NEG**: Negative result for serological determination and also for RT-PCR of SARS-CoV-2. Significance of the differences between groups were assessed by Kruskal-Wallis test followed by a post-hoc Conover's Test of multiple comparisons. **p<0.05, ** p<0.01, ***p<0.001, **** p<0.0001*


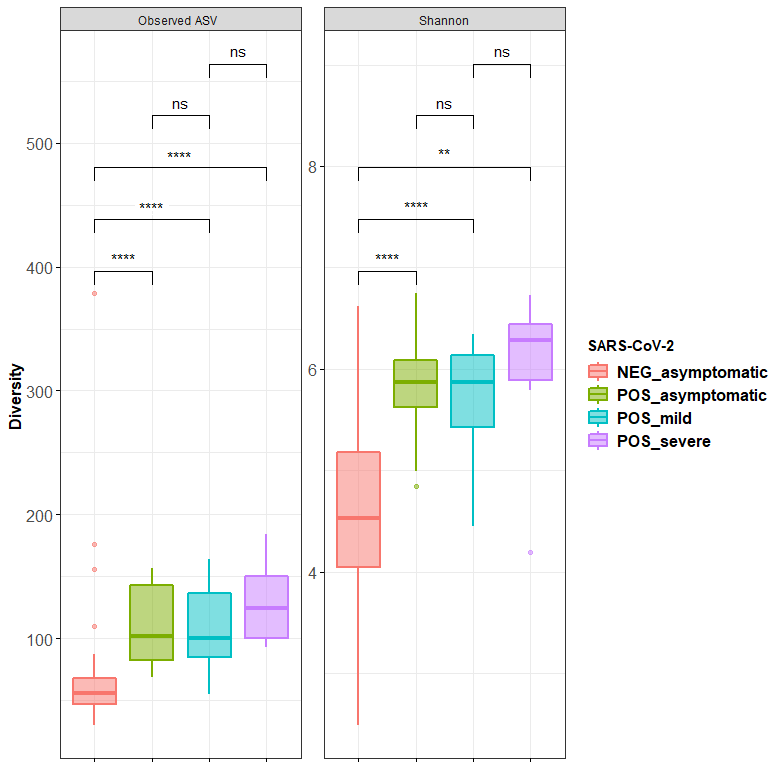


**Figure S4.** Nasopharyngeal microbiota alpha diversity measured as Observed ASV (richness) and Shannon (diversity) index according to the severity of the SARS-CoV-2 infection symptomatology. Significance of the differences between groups were assessed by Kruskal- Wallis test followed by a post-hoc Conover's Test of multiple comparisons. **p<0.05, ** p<0.01, ***p<0.001, **** p<0.0001.*
